# Age Differences in the Interpretation of Facial Emojis: Classification on the Arousal-Valence Space

**DOI:** 10.3389/fpsyg.2022.915550

**Published:** 2022-07-14

**Authors:** Gaku Kutsuzawa, Hiroyuki Umemura, Koichiro Eto, Yoshiyuki Kobayashi

**Affiliations:** Human Augmentation Research Center, National Institute of Advanced Industrial Science and Technology, Kashiwa, Japan

**Keywords:** emojis, arousal, valence, core affect, age differences, emotional states

## Abstract

Emojis are universal tools that are frequently used to express people’s emotional states throughout daily communications. They are often applied in various fields of research, such as consumer surveys, as indicators of users’ emotional states. Further analyses of emoji interpretation among people with age are required to ensure the validity of emojis as a metric in such fields of research, thereby reducing misunderstandings. However, details regarding the effect of age on both arousal and valence, as they pertain to the interpretation of emojis, remain unclear. Therefore, in this study, we investigate the effects of the interpretation of facial emojis on the arousal-valence space among people of varying age groups. We conducted an online survey involving 2,000 participants, whereby we employed a nine-point scale to evaluate the valence and arousal levels associated with 74 facial emojis. Based on the two axes of valence and arousal among the age groups involved in this study, emojis are categorized into six similar clusters. For the two negative clusters, i.e., strongly negative and moderately negative sentiments, the group involving middle-aged participants showed significantly higher levels of arousal compared to the group involving young participants. Additionally, not all emojis classified into the aforementioned negative clusters indicate age difference. Based on these results, this study recommends using emojis with no age-related effects on the negative clusters as indices for evaluating human emotions.

## Introduction

Across the world, people frequently use emojis to express their emotional states throughout their daily communications. Scientifically, emojis are associated with a variety of human emotions (Jaeger et al., 2019; Kutsuzawa et al., 2022). Emojis have also been applied in various fields of research, such as consumer studies, to assess users’ emotional states. For example, in a study conducted by Schouteten et al. (2018), a set of human facial emojis was used to investigate participants’ attitudes toward specific food products and evaluate their preferences toward such products. Because the use of emojis is not limited to a specific age or language, emojis can be used as universal indicators of emotional states, thereby expanding their range of application. However, the interpretation of emojis may be affected by age. Therefore, further analyses on how interpretations of emojis differ among people of different age groups are required.

The arousal levels of the emotions people perceive through media change with age. For example, Gilet et al. (2012) assessed 835 French adjectives, and they reported that middle-aged and older adults tend to perceive such adjectives with higher levels of arousal compared to young people. Similarly, Trnka et al. (2021) reported that middle-aged and older adults interpret them as higher levels of arousal from words that express emotions, such as, anger, fear, sadness, happiness, disgust, hope, love, and hate, compared to young people. Furthermore, Gavazzeni et al. (2008) used images from the International Affective Picture System (IAPS) to prove that middle-aged and older adults interpret them as higher levels of arousal than young people when they see specific images. These findings suggest that the arousal levels among such individuals may increase when they interpret emojis. Recently, Weiß et al. (2020) investigated the effect of age on emoji interpretation. However, they only assessed this effect on the valence level. Arousal and valence are two independent axes used to plot human emotional states, i.e., the core affect (Russell and Barrett, 1999). Facial emojis can be plotted on these two axes as well (Kutsuzawa et al., 2022). Therefore, understanding the effect of age on emoji interpretation, as it pertains to both arousal and valence levels, is crucial to ensure the validity of using emojis as a metric for consumer studies involving people of different age groups.

In this study, we investigate the interpretation of facial emojis and their classification in the arousal-valence space among people of varying age groups. As mentioned above, previous studies have reported that middle-aged people perceive higher levels of arousal from media compared to young people. Therefore, this study compares the interpreted arousal and valence levels between young and middle-aged people. Based on the findings of previous studies (Gavazzeni et al., 2008; Gilet et al., 2012; Trnka et al., 2021), this study hypothesizes that, compared to young people, middle-aged people interpret significantly higher arousal levels than young people from facial emojis as well as other media used in previous studies.

## Materials and Methods

### Participants

We conducted an online survey in the capital of Japan until 1,000 valid responses (from 500 males and 500 females) were obtained for each age group. The group comprising individuals aged 20–39 years was defined as young, whereas the group comprising individuals aged 40–59 years was defined as middle-aged to avoid bias by age group or gender. A total of 2,314 participants (young males: 595, females: 559, *M* age = 30.20, *SD* = 5.68. middle-aged males: 596, females: 564, *M* age = 49.40, *SD* = 5.43) participated in the survey. The participants were registered on an online panel maintained by a marketing research firm.^1^ They were also fluent in Japanese (the language used in the survey). Ethical approval was obtained before data collection, and the eligible participants were informed about data confidentiality. Informed consent was obtained before participation in the study. All study protocols were reviewed and confirmed by the local institutional review board (Committee on Ergonomic Experiments of the National Institute of Advanced Industrial Science and Technology).

### Emojis Used in This Study

This study employed human facial emojis, similar to previous studies, such as those conducted by Jaeger et al. (2019) and Kutsuzawa et al. (2022), because they were the most frequently used categories of emojis. Out of 89 facial emojis registered on Twemoji (2014), 74 emojis were selected and 15 excluded (e.g., 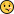, 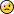 owing to the difficulty in explaining their emotions during the preliminary survey (See the Supplementary Material for more information on the preliminary survey). Because emoji designs slightly differ across services (i.e., Twitter, Instagram, Facebook, and WhatsApp), the study used the emoji designs displayed on Twitter. The emojis were saved as an image file and displayed on an appropriately sized screen (2.16 cm × 2.16 cm) to ensure that the participants could observe them clearly. Additionally, to avoid influencing the evaluations, no information other than that of the images (e.g., labels for emojis) was included.

### Questionnaire

The online questionnaire comprised two parts. The first examined participants’ socio-demographic and background characteristics. The second evaluated the arousal and valence levels associated with each emoji, similar to previous studies (Jaeger et al., 2019; Kutsuzawa et al., 2022). Specifically, following the lead sentence [“Please tell us your intended emotional state when using the following emojis in daily life (messages, social networking, etc.)”], respondents were asked to rate the presented emojis on a 9-point scale for valence (“Do you think the emotions indicated by the emojis are pleasant or unpleasant?” with 1 representing displeasure and 9 representing pleasure) and arousal (“How much emotional intensity do you think emojis express?” with 1 representing weak and 9 representing strong). Considering participants’ workload, they were asked to rate only 30 of the 74 emojis individually. There were 16 different pre-defined patterns for the order in which the emojis were presented, and each participant was randomly assigned one pattern to ensure that the number of respondents did not differ by age group. Consequently, each emoji was assessed by a minimum of 750 participants.

To determine whether participants answered the questions properly, two dummy questions were included after every 10 questions (i.e., for questions 11 and 21). These questions could be easily answered if the instructions were understood (e.g., “What is the subject of this questionnaire that you are being asked to answer?”). Participants who could not answer these questions correctly were excluded from further analyses.

### Data Analysis

To understand the effect of age on the interpretation of various emojis within the arousal-valence space, hierarchical cluster analyses and two-way analysis of variance (ANOVA) were conducted. Specifically, hierarchical cluster analysis was conducted to investigate the way in which the 74 emojis were classified into several clusters. Two-way ANOVA was conducted to reveal the characteristics of each cluster and the differences in interpretation by the age groups. The Bonferroni correction was used for a *post hoc* analysis after the main effect was obtained. The results of the ANOVA were considered statistically significant if the *p*-value was less than 0.05. Furthermore, to clarify the specific emojis that were interpreted differently between the age groups, independent-samples *t*-tests were conducted on all emojis classified into clusters with main effects. The results of the *t*-tests were considered statistically significant if the *p*-value was less than 0.05. Statistical analyses were conducted mainly using IBM SPSS Statistics 26 (SPSS Inc., Chicago, IL, United States), and R software (R Core Team, 2016) was used only for the Calinski-Harabasz index, which cannot be analyzed using SPSS.

## Results

### Analyzed Data

**FIGURE 1:**
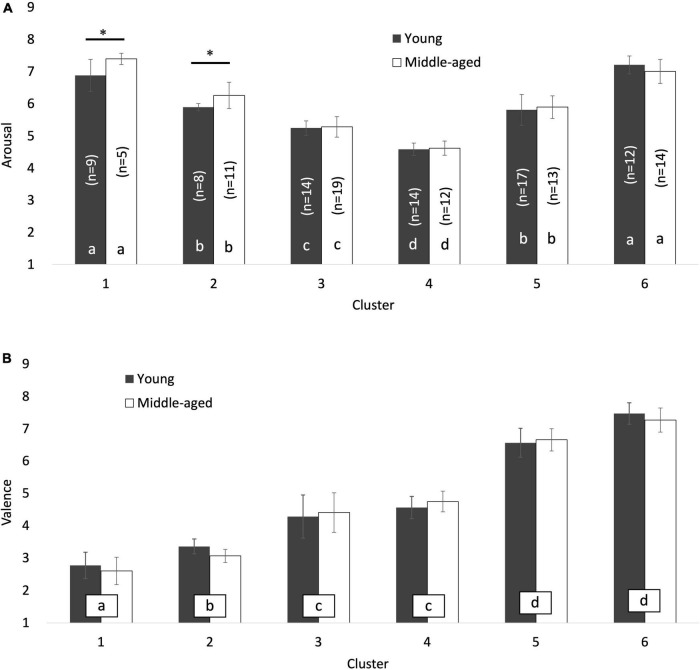
The core affect overlaid for each cluster on the scatter plot of the mean arousal and valence scores for 74 facial emojis in **(A)** young people and **(B)** middle-aged people. The vertical and horizontal axes represent the valence and arousal levels, respectively. The error bars indicate one standard deviation for each variable (i.e., valence and arousal). Each colored circle represents the range of ±1 standard deviation from the mean emotional valence and mean arousal for each cluster. The brackets in the bars indicate the number of emojis belonging to each cluster, which is equal in panels **(A,B)**. Asterisks indicate significant differences (*p* < 0.05).

The responses to the two dummy questions were examined, and 314 participants who responded incorrectly to both were excluded from the remaining analyses. Consequently, the data obtained from 2,000 participants could be employed for further analyses (young males: 500, females: 500, *M* age = 30.13, *SD* = 5.55. middle-aged males: 500, females: 500, *M* age = 49.71, *SD* = 5.44). The mean and standard deviation of the arousal and valence levels for each emoji per age group were plotted on a scatter plot (Figures 1A,B). In this plot, the horizontal and vertical axes denote the arousal and valence levels, respectively.

### Emoji Classification on the Arousal-Valence Space for Each Age Group

**FIGURE 2:**
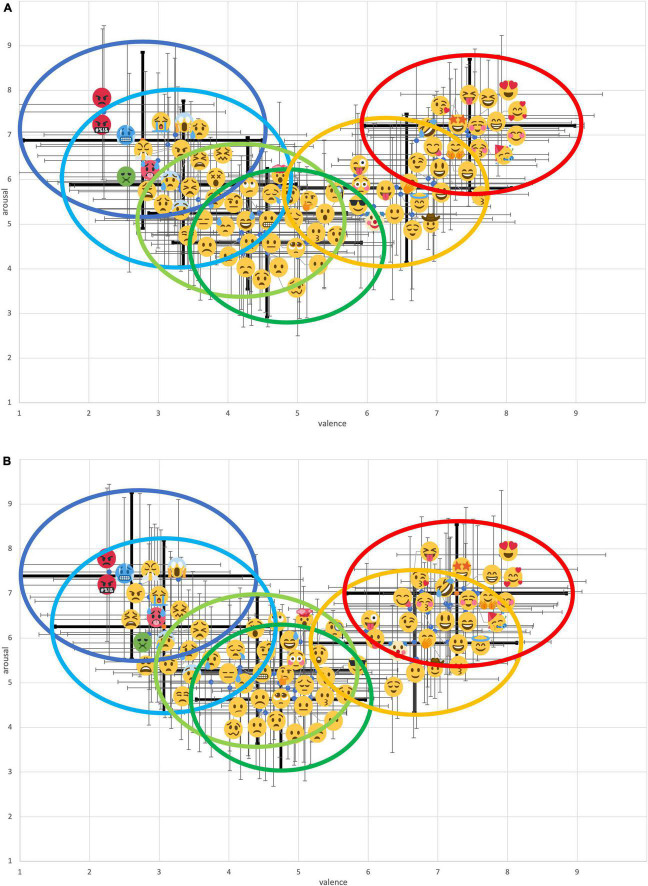
Comparison of **(A)** mean arousal and **(B)** mean valence scores for each cluster per age group. Error bars are ±1 standard deviation of the mean. Asterisks indicate significant differences between age groups. Each letter indicates *post hoc* results (Bonferroni corrected) between clusters, with clusters of similar letters being not significantly different at 5%. The “*n*” in the bar chart represents the number of emojis belonging to each cluster.

Hierarchical cluster analyses were performed on the mean arousal and valence data of each age group (the young group comprises individuals aged 20–39 years and the middle-aged group comprises individuals aged 40–59 years) to classify similar emojis into several clusters. The Euclidean distance and Ward aggregation criterion were considered in the analyses (Z-scores were calculated and used for each rating). The optimum number of clusters was obtained from the dendrogram and the Calinski-Harabasz index. As a result, a six-cluster solution was retained for both the young and middle-aged groups. The mean (±standard deviation) for arousal and valence scores and the number of emojis classified into each cluster are displayed in Figures 2A,B, respectively.

### Effect of Age on Emoji Interpretation

A two-way ANOVA was performed on the mean arousal and mean valence for each emoji to analyze the effects of age group and cluster. For arousal, a significant main effect was found on the clusters [*F*(5, 136) = 203.07, *p* < 0.001, η^2^ = 0.88] and the age [*F*(1, 136) = 5.65, *p* < 0.05, η^2^ = 0.04]. Furthermore, significant interactions between clusters and age were found [*F*(5, 136) = 2.73, *p* < 0.05, η^2^ = 0.09]. After the interaction between clusters and age was found, the simple main effects on both clusters and age were analyzed. Consequently, for clusters 1 and 2 only (*p*s < 0.05), the middle-aged group indicated significantly higher arousal scores than the young group. For valence, a significant main effect was found on the clusters [*F*(5,136) = 386.76, *p* < 0.001, η^2^ = 0.93] but not on age [*F*(1, 136) = 0.317, *n.s.*] and the interaction between clusters and age [*F*(5,136) = 1.24, *n.s.*].

To determine specific emojis affected by age group difference and identify emojis with an age-related effect on the arousal level, independent-sample *t*-tests were conducted on each of the 17 emojis classified into clusters 1 and 2. The results demonstrated significant differences on three emojis, as listed in Table 1, and no significant differences on 14 emojis, as listed in Table 2.

**TABLE 1:** Negative emojis show differences in arousal levels by age group per cluster for strongly negative and moderately negative sentiments.

NAME	EMOJI	Age-group	N	Arousal		
Face with steam from nose	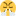	Young	437	6.75	(1.98)	**
		Middle-aged	438	7.18	(2.05	
Confounded face	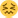	Young	437	5.86	(1.89)	**
		Middle-aged	438	6.42	(2.01)	
Tired face	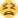	Young	437	6.06	(2.01)	**
		Middle-aged	438	6.52	(1.95)	

The emoji names are registered on Twemoji. The figures in parentheses are standard deviations. Asterisks indicate significant differences (*p < 0.05).

**TABLE 2:** Emojis showing no difference in arousal level by age group per clusters for strongly negative and moderately negative sentiments.

NAME	EMOJI	Age-group	N	Arousal	
Angry face	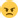	Young	375	6.54	(1.89)
		Middle-aged	375	6.83	(1.73)
Pouting face	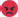	Young	375	7.69	(1.69)
		Middle-aged	375	7.65	(1.72)
Face with symbols on mouth	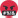	Young	375	7.52	(1.94)
		Middle-aged	375	7.48	(1.96)
Face screaming in fear	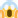	Young	437	7.00	(2.04)
		Middle-aged	438	7.32	(1.79)
Loudly crying face	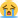	Young	376	6.78	(2.05)
		Middle-aged	374	6.92	(2.07)
Nauseated face	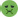	Young	376	6.16	(2.23)
		Middle-aged	374	6.08	(2.03)
Hot face	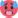	Young	375	6.43	(1.96)
		Middle-aged	375	6.52	(1.95)
Cold face	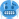	Young	375	7.07	(1.98)
		Middle-aged	375	7.35	(1.80)
Worried face	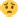	Young	375	5.73	(1.72)
		Middle-aged	375	5.69	(1.80)
Persevering face	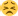	Young	437	5.85	(1.77)
		Middle-aged	438	6.08	(1.86)
Weary face	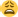	Young	437	5.93	(1.92)
		Middle-aged	438	5.89	(1.92)
Anxious face with sweat	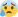	Young	437	5.96	(1.96)
		Middle-aged	438	6.13	(1.91)
Sad but relieved face	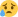	Young	375	5.76	(1.82)
		Middle-aged	375	5.77	(2.04)
Dizzy face	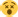	Young	376	5.98	(1.89)
		Middle-aged	374	6.16	(1.71)

*The names of the emojis are those registered on Twemoji. The figures in parentheses are standard deviations.*

## Discussion

This study aimed to understand the age differences of facial emoji interpretations on the arousal-valence space. To this end, data obtained from 2,000 participants were collected, and the arousal and valence levels indicated using 74 emojis were analyzed. For both the young and middle-aged groups, emojis were distributed in a U-shape on the two axes and categorized into six clusters (Figure 1). These trends were similar to the results of previous studies, such as those conducted by Jaeger et al. (2019) and Kutsuzawa et al. (2022). Therefore, each cluster can be interpreted as follows: cluster 1, a strongly negative sentiment cluster; cluster 2, a moderately negative sentiment cluster; cluster 3, a neutral sentiment cluster with a negative bias; cluster 4, a neutral sentiment with a low arousal cluster; cluster 5, a moderately positive sentiment cluster; and cluster 6, a strong positive sentiment cluster. As expected, the effect of age was found only in the arousal levels of negative clusters (i.e., clusters 1 and 2). The middle-aged group tended to indicate significantly higher arousal levels than the young group for both clusters. These results suggest that middle-aged people tend to interpret stronger levels of arousal from negative emojis compared to young people, thereby supporting part of our initial hypothesis.

Our interpretation of human facial emojis, especially negative ones, may be influenced by the changes in the way we interact with society as we age. This study revealed that middle-aged people tended to interpret the emojis classified into two negative clusters (i.e., strongly negative and moderately negative sentiment clusters) with stronger arousal levels than young people, which is in line with the findings of Weiß et al. (2020). This phenomenon was especially prominent in the case of the following three emojis: 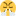, 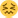, and 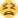 (Tables 1, 2). According to Emojipedia (2016), the “face with steam from nose 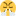 ” expresses both negative and positive emotions such as “contempt,” “frustration,” “anger,” “pride,” “superiority,” and “power.” Furthermore, Sick et al. (2020) indicate that the “confounded face 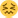
” and “tired face 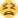
” express “unhappiness,” “disappointment,” and “guilt.” Interestingly, middle-aged people reported stronger arousal levels than young people for the words “contempt” and “guilt” as well (Trnka et al., 2021). It has been reported that people generally become more sensitive to the stimulus that evokes negative emotions as they get older (Trnka et al., 2021). Orth et al. (2010) suggested that this tendency is a result of the prosocial and adaptive interpersonal behaviors required when aging. Therefore, the phenomenon of middle-aged people perceiving stronger arousal levels from emojis compared to young people can be considered to be influenced by the changes in the way people interact with society.

The interpretation of emojis used as the index for measuring emotion should be consistent among different demographics. This study found that middle-aged people evoke stronger levels of arousal for negative emojis than young people. This implies that negative emotions may not be captured stably by emojis among different age groups. As mentioned above, this trend was especially prominent in the following three emojis: 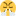, 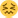, and 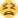 (Tables 1, 2). Contrarily, no significant differences were observed across age groups on the level of arousal for the following emojis: 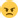, 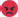, 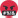, 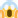, 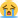, 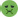, 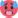, 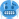, 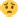, 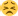, 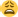, 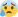, 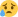, and 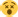. Based on the results, this study suggests using these 14 emojis for negative clusters when using emojis as indices to measure human emotions across various age groups.

This study has several limitations that must be acknowledged when interpreting the results. First, all participants in this study were Japanese adults, and individuals with other demographic characteristics, such as gender and culture, were not included. Although the “use” of emojis has been reported to be significantly influenced by demographic characteristics, such as gender and culture (Bai et al., 2019), their “interpretation” has been considered not significantly impacted by these characteristics (Kralj Novak et al., 2015; Jaeger et al., 2018, 2019). Therefore, the results of this study are consistent with other demographic characteristics.

Second, only the emojis displayed on Twitter were used in this study. Therefore, slight differences in emoji designs may affect the results. For example, the design of emojis displayed on Android and iPhone devices differ slightly, even though the same code is used. These differences in design have been shown to affect the interpretation of emojis, especially for emojis that express ambiguous emotions (Rodrigues et al., 2018). Therefore, the results of this study may differ when other emoji designs are used, but the specific differences that may occur are unclear. Therefore, these differences should be clarified when interpreting emojis across designs.

Finally, the study clarified the overall trend in the interpretation of emojis by age group. However, the same participant may interpret emojis differently depending on the timing of responses (Robertson et al., 2021). Significantly, intra-individual variations in the interpretation of emojis have not been discussed thus far. To use emojis as indicators of participants’ emotional states in psychology and consumer research, it is crucial to understand which emojis are interpreted stably, not only by age but also by individuals.

This study attempted to understand age differences (i.e., young and middle-aged people) and emoji interpretations on the arousal-valence space. The results demonstrated the following: (1) emojis were categorized into six similar clusters on the two axes of valence and arousal for both age groups, (2) the middle-aged group tended to indicate significantly higher levels of arousal than the young group for two negative clusters (i.e., strongly negative and moderately negative sentiments), and (3) not all emojis classified into these two negative clusters indicated age differences and only three emojis (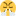, 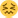, and 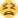) showed differences in interpretation among different age groups. Based on the results, this study suggests using emojis with no age effects for negative clusters when using emojis as indices for measuring human emotions among people of various ages.

## Data Availability Statement

The raw data supporting the conclusions of this article will be made available by the authors, without undue reservation.

## Ethics Statement

Ethical review and approval was not required for the study on human participants in accordance with the local legislation and institutional requirements. The patients/participants provided their written informed consent to participate in this study.

## Author Contributions

GK and YK performed the experiments and drafted the manuscript. GK analyzed the data. HU, KE, and YK provided guidance and oversight. GK and YK drafted the manuscript. All authors contributed notes and edits within the manuscript and provided critical feedback for improving the manuscript.

## Conflict of Interest

The authors declare that the research was conducted in the absence of any commercial or financial relationships that could be construed as a potential conflict of interest.

## Publisher’s Note

All claims expressed in this article are solely those of the authors and do not necessarily represent those of their affiliated organizations, or those of the publisher, the editors and the reviewers. Any product that may be evaluated in this article, or claim that may be made by its manufacturer, is not guaranteed or endorsed by the publisher.

## Footnotes

1
https://candc.co.jp/
