# Supplementary material for: Age Differences in the Interpretation of Facial Emojis: Classification on the Arousal-Valence Space
Source: Front Psychol. 2022 Jul 14;13:915550. doi: 10.3389/fpsyg.2022.915550 (PMC9333063; doi:10.3389/fpsyg.2022.915550)
Supplement: Supplementary file 1 [file Data_Sheet_1.pdf]

A preliminary study was conducted to extract emojis that indicate emotional states from the 89 types of face pictograms registered on Twemoji (as of August 2020). The survey was conducted on 26 staff members of the authors' institutions (males: 9, females: 17, M age = 43.62, SD = 10.62). Participants were asked to choose from the nine subscales (i.e., Depression (Anxiety), Hostility, Boredom, Liveliness, Well Being, Friendliness, Concentration, and Startle) of the multiple mood scale (Terasaki et al., 1992) or "N/A" regarding the emotional state the presented emoji represented. Considering participants' workload, they were asked to rate only 30 of the 89 emojis at random. Consequently, each emoji was assessed by a minimum of 6 participants. As a result, "N/A" was calculated as the mode for 15 emojis (e.g., 🤒, 🤕). Based on the results, the present survey was conducted on a subset of 74 emojis, excluding these 15 emojis.
